# IL11 signaling mediates piR-2158 suppression of cell stemness and angiogenesis in breast cancer

**DOI:** 10.7150/thno.82538

**Published:** 2023-04-17

**Authors:** Qian Zhao, Lu Qian, Yuefan Guo, Jinhui Lü, Danni Li, Heying Xie, Qiong Wang, Wenjing Ma, Pengfei Liu, Yu Liu, Tao Wang, Xuebiao Wu, Junyi Han, Zuoren Yu

**Affiliations:** 1 Key Laboratory of Arrhythmias of the Ministry of Education of China, Research Center for Translational Medicine, Shanghai East Hospital, Tongji University School of Medicine, Shanghai, China.; 2 School of Basic Medicine, Jinzhou Medical University, Liaoning, China.; 3 Shanghai OE Biotech Co., Ltd., Shanghai, China.

**Keywords:** piR-2158, breast cancer, cancer stem cell, IL-11

## Abstract

Emerging evidence has indicated the aberrant expression of PIWI-interacting RNAs (piRNAs) in human cancer cells to regulate tumor development and progression by governing cancer cell stemness. Herein, we identified downregulation of piR-2158 in human breast cancer tumors, especially in ALDH+ breast cancer stem cells (BCSCs) from patients and cell lines, which was further validated in two types of genetically engineered mouse models of breast cancer (MMTV-Wnt and MMTV-PyMT). Enforced overexpression of piR-2158 in basal-like or luminal subtypes of breast cancer cells suppressed cell proliferation, migration, epithelial-mesenchymal transition (EMT) and stemness *in vitro*. Administration of a dual mammary tumor-targeting piRNA delivery system in mice reduced tumor growth *in vivo*. RNA-seq, ChIP-seq and luciferase reporter assays demonstrated piR-2158 as a transcriptional repressor of *IL11* by competing with AP-1 transcription factor subunit FOSL1 to bind the promoter of *IL11*. STAT3 signaling mediated piR-2158-IL11 regulation of cancer cell stemness and tumor growth. Moreover, by co-culturing of MDA-MB-231 and HUVECs *in vitro* and CD31 staining of tumor endothelial cells *in vivo,* we demonstrated inhibition of angiogenesis by piR-2158-IL11 in breast cancer. In conclusion, the current study not only reveals a novel mechanism through which piR-2158 inhibits mammary gland tumorigenesis *via* regulating cancer stem cells and tumor angiogenesis, but also provides a novel therapeutic strategy in treatment of breast cancer.

## Introduction

P-element-induced wimpy testis (PIWI)-interacting RNAs (piRNAs) are a class of small non-coding RNAs with ~30 nt in length, which were highly enriched and first discovered in germ cells 1. piRNAs bind to PIWI family proteins to form piRNA-induced silencing complexes (piRISC), playing important roles in control of DNA stability, transposon transcription, heterochromatin formation and epigenetic regulation 2,3. Emerging evidence indicates aberrant expression of piRNAs and PIWI proteins in human cancer cells, especially in cancer stem cells, to regulate tumor initiation and progression 3-5. However, the regulatory mechanisms are yet to be determined.

Our previous small RNA-seq screening study identified a group of piRNAs in breast cancer cells, and demonstrated their involvement in regulation of breast cancer stem cell (CSCs). For example, we demonstrated the regulation of CSCs by piR-016658 in the estrogen receptor (ER) negative basal-like subtype of breast cancer 5, and by piR-823 in the ER positive luminal subtype of breast cancer 6. We found that piR-2158 was downregulated independently of the ER status in human breast cancer. PiR-2158 has been reported to involve in regulations of stem cells such as bone marrow-derived mesenchymal stromal/stem cells (MSCs) 7 and human cancer such as lung adenocarcinoma (LUAD) 8. Whether it regulates CSCs remains unknown. Herein, we validated the aberrant expression of piR-2158 in breast cancer patients, and applied for further analysis in breast CSCs.

CSCs are a small population of cancer cells with high heterogeneity and strong ability to regenerate tumors. In breast cancer, CSCs were identified using biomarkers CD24^low^CD44^high^ and/or aldehyde dehydrogenase 1 (ADLH1) positive 9,10. CSCs are responsible for tumor progression, metastasis, recurrence, and chemoresistance 11.

Angiogenesis is an essential step in tumor development and progression, and is required for invasive tumor growth and distant metastasis 12,13. Angiogenesis provides oxygen, nutrients and growth factors for tumor growth. Targeted inhibition of angiogenesis has been considered as an effective approach to suppress tumor growth. For example, monoclonal antibodies or small molecule tyrosine kinase inhibitors targeting vascular endothelial growth factor (VEGF) or its receptors 14 have showed high therapeutic efficiency in treatment of cancer.

Our current study confirmed downregulation of piR-2158 in breast cancer. Overexpression of piR-2158 inhibited cell proliferation, migration, invasion and stemness *in vitro* and suppressed tumorigenicity and angiogenesis *in vivo*. The mechanism study demonstrated significant inhibition of *IL11* expression by piR-2158 *via* competing with AP-1 transcription factor subunit FOSL1 to bind the promoter of *IL11*. Downregulation of IL-11 inactivated the JAK-STAT signaling pathway, leading to suppression of tumorigenesis. These findings not only reveal the regulatory mechanism of piR-2158 in suppressing breast cancer stem cells, but also suggest a novel therapeutic strategy in treatment of breast cancer.

## Methods and Materials

**Human breast tumor samples.** Human breast cancer tumor samples and adjacent normal tissues were collected from Tongji University Shanghai East Hospital. All the procedures were approved by the Institutional Review Board (IRB) of Shanghai East Hospital. All patients were provided with written informed consent form.

**Animals.** 6-week-old female nude mice and BALB/c mice were purchased from the Silaike Animal Company (Shanghai, China) for *in vivo* assays. Mammary tumors from MMTV-Wnt or MMTV-PyMT transgenic mice were prepared by Dr. Suling Liu's lab. All animal studies were approved by the Institutional Animal Care and Use Committee of the Tongji University School of Medicine.

**Cell lines and cell culture.** Human breast cancer cell line MDA-MB-231, mouse breast cancer cell line 4T1, human umbilical vascular endothelial cells (HUVECs) and human embryonic kidney 293T (HEK293T) cells were originally purchased from ATCC and maintained in our lab, and cultured in dulbecco's modified eagle medium (DMEM) or endothelial cell medium (ECM) supplemented with 10% fetal bovine serum (FBS), 1% penicillin/streptomycin and 1% endothelial cell growth when needed. Lung metastatic MDA-MB-231 sublines 4173 and 4175 were presented by Dr. Guohong Hu at the Shanghai Institutes for Biological Sciences, Chinese Academy of Sciences. All of these cells were cultured at 37 ℃ in a humidified environment with 5% CO_2_.

**Oligos and vectors.** All oligos for small RNAs and DNA primers were synthesized by GenScript (Nanjing, China). Oligo transfection was performed using RNAiMAX (Invitrogen) following the manufacturer's instructions with a final concentration of 30 nM. Lentivirus vector LV3(H1/GFP&Puro)-piR-2158 was purchased from GenePharma (Shanghai, China), using a sequence not homology to any known mammalian gene as negative control (NC). The FOSL1 coding sequence was amplified from the cDNA of MDA-MB-231 cells and ligated into pcDNA3.1(+) at the BamHI and HindIII enzyme digestion sites. All target sequences are listed in Table S1.

**ALDH assay.** Single cell suspensions were prepared from breast cancer tumor following a protocol described in our previous publication 15. ALDEFLUOR Kit (01700, STEMCELL, CA) was used for ALDH+ breast cancer stem cell isolation following the manufacturer's instruction.

**Cell Counting Kit-8 (CCK-8) assay.** 1x10^3^ cells/well were seeded into 96-well plates in eight repeats. After incubation for the indicated time, the cells were stained with 10 µL CCK-8 solution per well for 3 h under cell culture conditions, followed by the OD measurement at 450 nm.

**Wound healing assay.** 2x10^4^ cells/well were seeded into 12-well plates in triplicates. After the cell density reached 95%, FBS-reduced DMEM medium (0.1% FBS) was applied to starve the cells for 24 h, followed by creating a vertical wound in each well using a 10 µL pipette tip. The wound widths were photographed and quantified under microscope (Zeiss) at the indicated time points. Six fields were randomly selected for statistical analysis using Image J software.

**Cell invasion assay.** Transwell chambers with 8-μm pores (#353097, Corning Falcon) were pre-coated with Matrigel (#356231, Corning), and placed in a 24-well plate (#353504, Corning Falcon) containing cell culture medium. 2 x 10^4^ pre-starved cells were seeded in the chambers with serum-free medium, followed by 6 hours' incubation at 37 °C and 5% CO_2_. Cells adherent to the upper surface of chambers were removed using cotton swabs. Chambers containing invaded cells were stained with 0.4% violet crystal acetate overnight. Six fields were randomly selected for photography using a microscope (Zeiss). The number of invaded cells was counted for statistical analysis.

**Quantitative real-time PCR (qRT-PCR) analysis of piRNA and mRNA**. Quantitative analysis of piRNAs and mRNAs were performed following our previous publication 6. 5s ribosomal RNA was used for normalization of piRNAs. GAPDH or β-actin were used for normalization of mRNAs. The primer sequences are listed in Table S2. All primers were synthesized by GenScript (Nanjing, China).

**Western blot assay.** The following primary antibodies were used for western blot (1:1,000 dilution): anti-Slug (sc-166476, Santa Cruz), anti-Vimentin (sc-32322, Santa Cruz), anti-Fibronectin (sc-8422, Santa Cruz), anti-ZEB1 (sc-515797, Santa Cruz), anti-KLF4 (ARG-55811, Arigo Inc.), anti-NANOG (4903S, Cell Signaling Technology), anti-SOX2 (3579, Cell Signaling Technology), anti-OCT4 (2750S, Cell Signaling Technology), anti-IL-11 (55169-1-AP, Proteintech), anti-STAT3 (4904, Cell Signaling Technology), anti-Phospho-STAT3 (Tyr705) (9145T, Cell Signaling Technology), anti-GAPDH (sc-47724, Santa Cruz) and anti-β-actin (sc-47778, Santa Cruz). HRP-linked anti-rabbit IgG (7074S, Cell Signaling Technology) and HRP-linked anti-mouse IgG (7076S, Cell Signaling Technology) were used as secondary antibodies (1:5,000 dilution). Three independent experiments were performed for statistical analysis.

**Differently expressed genes (DEGs) screening and pathway enrichment analysis.** MDA-MB-231 cells with or without overexpression of piR-2158 (n = 3 in each group) were applied RNA-seq analysis using BGISEQ sequencing platform. The abundance of each gene was quantified as TPM (Transcripts per million) value for differential analysis. 107 differently expressed genes (DEGs) were identified using the absolute value of fold change (FC) greater than 2 and p-value less than 0.05 as cutoff values.

Functional enrichment analysis of 107 differently expressed genes (DEGs) was performed by Over-Representation Analysis (ORA) with Kyoto Encyclopedia of Genes and Genomes (KEGG) pathway and Gene Set Enrichment Analysis (GSEA) with WikiPathway using web-based gene set analysis toolkit (WebGestalt).

**Enzyme linked immunosorbent assay (ELISA) for secretory IL-11.** Quantitative analysis of IL-11 in the supernatants of breast cancer cells was performed using the Human IL-11 ELISA Kit (70-EK111-96, MultiSciences, Hangzhou, China) following the manufacturer's instruction.

**Immunohistochemistry (IHC) & Immunofluorescence (IF) staining.** IHC and IF staining were performed following our previous publication 16. Anti-Ki67 (ab16667, Abcam) or anti-CD31 (ab28364, Abcam) was used as primary antibodies (1:200 dilution). HRP-linked goat anti-rabbit IgG (ab6721, Abcam) (1:500 dilution) was used as secondary antibody for the IHC analysis. Alexa Fluor® 488-linked goat anti-rabbit IgG (ab150077, Abcam) (1:500 dilution) was used as secondary antibody for the IF analysis. Cell nucleus were stained with 6-diamidino-2-phenylindole (DAPI) (D9542, Sigma). Leica DM3000 microscope was used for photography. Leica Application Suite X software 3.0.0.15697 was used for quantitative analysis. All the staining analyses were performed in triplicates. Three fields were randomly selected in each sample statistical analysis.

**HUVECs tube formation assay.** HUVECs were cultured using the conditioned medium from breast cancer cells MDA-MB-231 cells with or without overexpression of piR-2158. Exogenous recombinant human protein IL-11 (rHu IL-11, R&D) were added into the medium at a final concentration of 30 ng/mL as indicated. For the tube formation assay, precooled 96-well plates were coated with matrigel for 60 min in a cell culture incubator, then seeded with 3 × 10^4^ HUVECs in each well and cultured in the conditioned medium with or without addition of exogenous rHu IL-11. Tube formation was photographed using a microscope at 12 h intervals. Tube formation ability was quantified by measuring the cumulative tube length in five random fields under a microscope.

**Luciferase reporter assay for the* IL11* Promoter activity.** *IL11* promoter was amplified from the genomic DNA of MDA-MB-231 cells and ligated into pGL3-promoter vector (E1761, Promega) at the KpnI and XhoI enzyme digestion sites. 1 x 10^4^ HEK-293T cells were seeded in 24-well plates. When the cell density reached 80%, co-transfection was performed using 1 µg pGL3-promoter-IL-11, 1 µg pcDNA3.1(+)-FOSL1 and 20 pmol piR-2158 mimic per well. Lipofectamine 2000 was used as the transfection reagent. In 24 h after transfection, luciferase activities were measured using the Luciferase Reporter Assay Kit (E1910, Promega, USA).

**Preparation of mammary tumor mice.** 1 x 10^6^ 4T1 or MDA-MB-231 cells with or without overexpression of piR-2158 were mixed with matrigel and injected into the fat pad of the fourth mammary gland of BALB/c or nude mice (n = 10 for each group), respectively. From one week after cell transplantation on, the tumor volumes were measured every 2 days until the mice were sacrificed at the indicated timepoint. The tumor growth curves were plotted. All tumors were weighted and applied for further analysis.

**Administration of magnetic nanoparticles pre-loaded with piR-2158 or NC mimic (piR-2158/NC-MNPs).** Zn_0.4_Fe_2.6_O_4_@SiO_2_ nanoparticles were coated with hyaluronic acid (HA), pre-loaded with piR-2158 or NC mimic, and self-assembled following the published procedure described in our previous publication 5. In one week after the cancer cell transplantation, the mammary tumor mice were tail vain-injected with the piR-2158/NC-MNPs (1 mg/kg body weight per dose every two days). Immediately after each injection, a piece of magnet was placed near the tumor tissue for 1 h.

**Public data analysis.** TCGA database were used to analyze the gene expression levels and survivals of breast cancer patients using R packages through Xiantao online tools (https://www.xiantao.love/). For *IL11* survival curve analysis, surv_cutpoint function in survminer package was first used for optimal group cut-off screening. Then survival package was used to test the proportional risk hypothesis and fitted survival regression. The results were visualized using survminer package and ggplot2 package. ChIP-seq data of transcription factor FOSL1 in MDA-MB-231 and BT549 was obtained from Cistrome DB database (http://cistrome.org/db/#/) and analyzed using the online website (WashU browser, http://epigenomegateway.wustl.edu/browser/).

**Statistical analysis.** Data are presented as mean ± SEM unless otherwise stated. The two-tailed student's t test was used in statistical comparisons. p < 0.05 was considered statistically significant.

## Results

**Figure 1:**
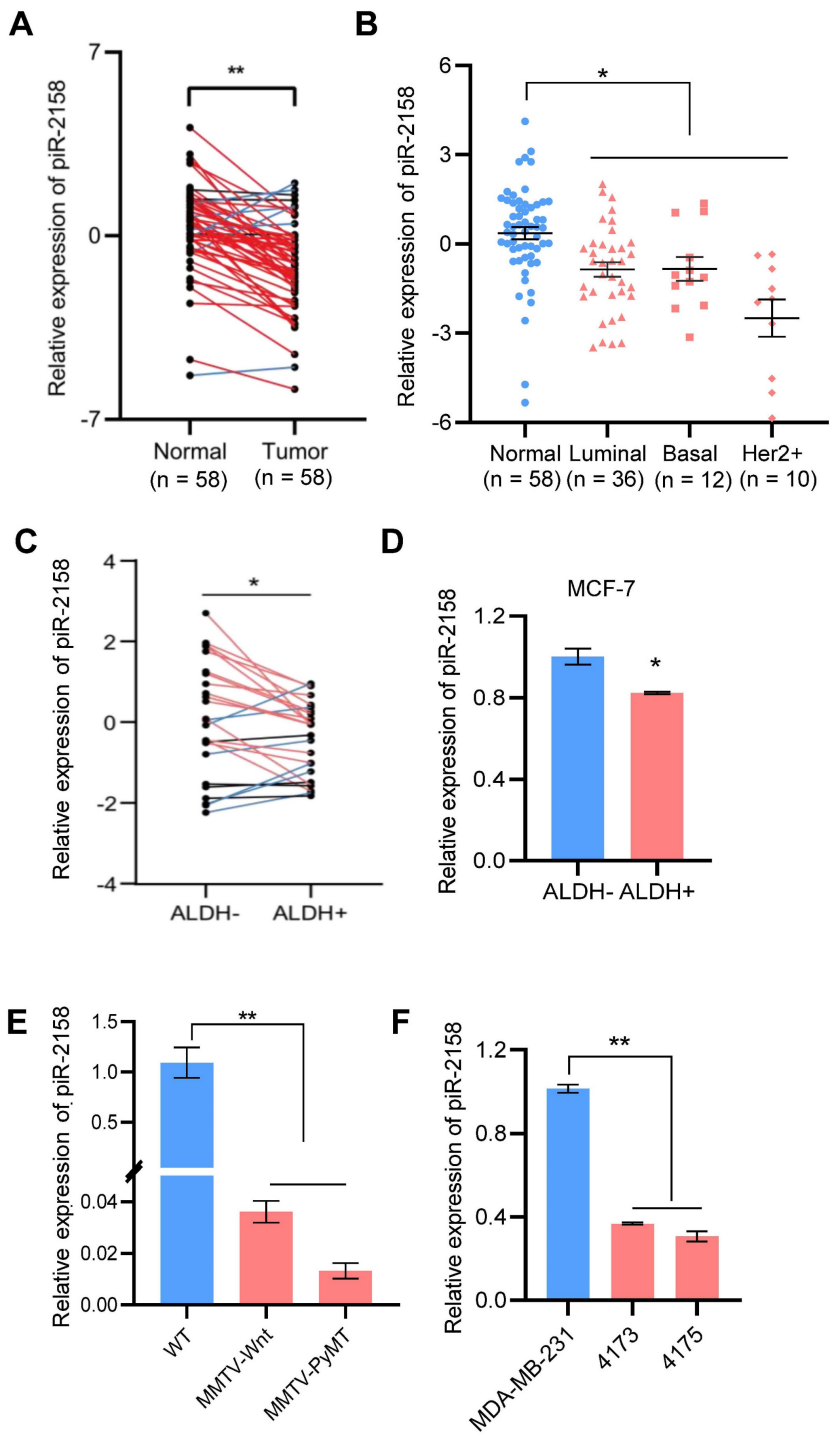
** Downregulation of piR-2158 in breast cancer.** A: Expression of piR-2158 in tumors and the matching adjacent non-malignant tissues from 58 breast cancer patients. B: Expression of piR-2158 in the 58 breast cancer patients grouped by the tumor subtypes (n = 58). C: Expression of piR-2158 in ALDH1+ breast CSCs isolated from breast cancer patients, compared to ALDH1- breast cancer cells (n = 25). D: Expression of piR-2158 in ALDH1+ breast CSCs isolated from MCF-7 breast cancer cells (n = 3). E: Expression of piR-2158 in the mammary tumors from MMTV-Wnt and MMTV-PyMT transgenic mice (n = 3). F: Expression of piR-2158 in MDA-MB-231 cells and its two lung-metastatic sublines 4173 and 4175 (n = 3). Data are presented as the mean ± SEM. * p < 0.05, ** p < 0.01.

**Downregulation of piR-2158 in human and rodent breast cancer tumors.** Our previous small RNA screening study indicated involvement of a group of piRNAs in regulation of breast cancer, including piR-2158 (Table S3) 5. In order to clarify the function of piR-2158, we firstly validated the expression characteristics of piR-2158 in breast cancer. 58 patients including 36 luminal, 12 basal-like and 10 Her2+ subtypes of breast cancer were applied for gene expression analysis. piR-2158 showed downregulation in primary tumors compared to the matching adjacent non-malignant tissues (Figure 1A), which was independent of the tumor subtypes (Figure 1B). Additional analysis on breast CSCs indicated downregulation of piR-2158 in aldehyde dehydrogenase 1 (ALDH1) positive breast CSCs, compared to ALDH1- differentiated breast cancer cells (Figure S1-S2, Figure 1C-1D). Moreover, two types of transgenic mouse models of PyMT- or Wnt-induced breast cancer further confirmed downregulation of piR-2158 in breast cancer tumors (Figure 1E). Two metastatic sublines of MDA-MB-231, 4173 and 4175, showed lower levels of piR-2158 than the parental cell line (Figure 1F).

**Figure 2:**
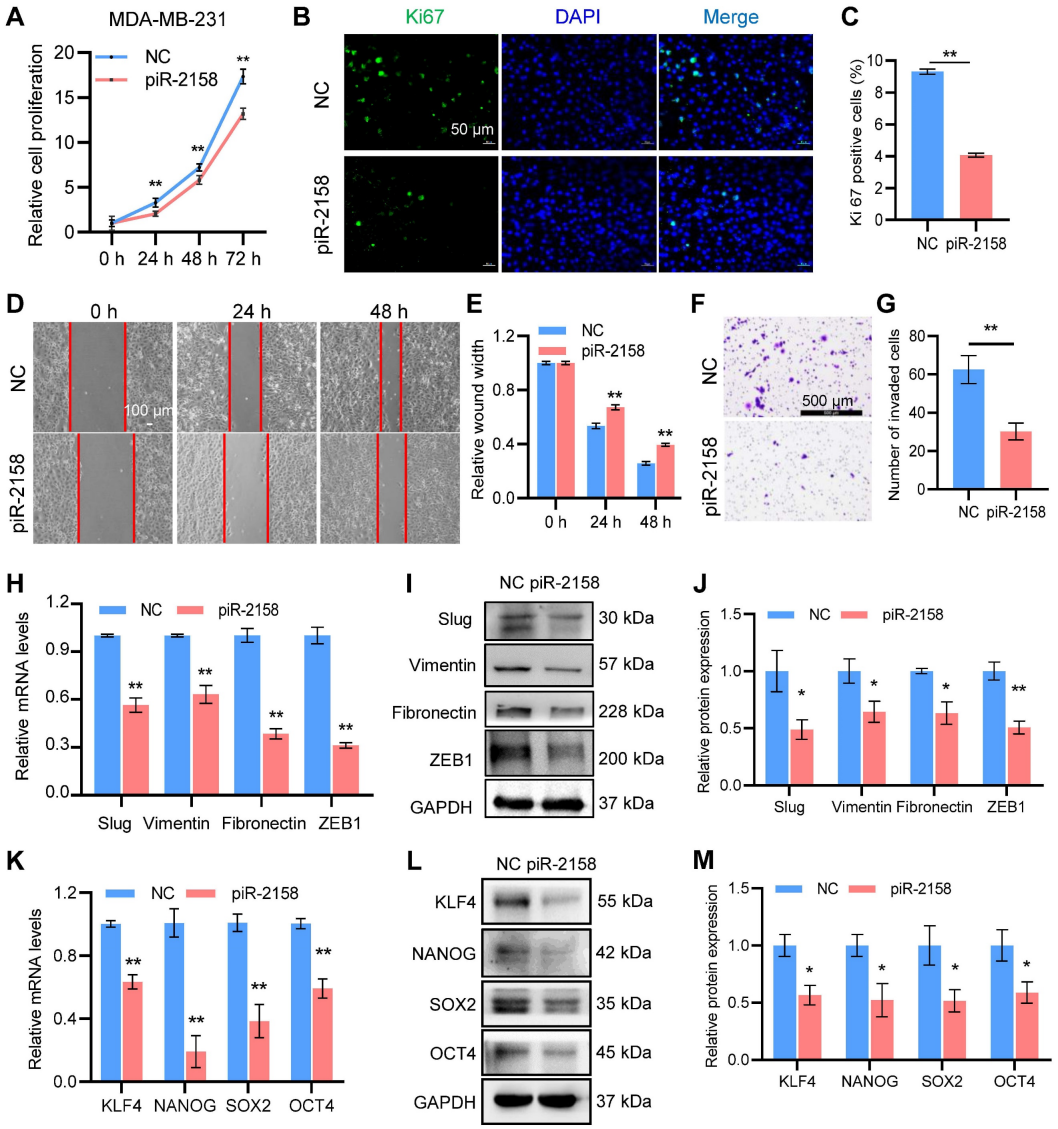
** piR-2158 suppressed cell proliferation, migration, EMT and stemness in human breast cancer.** A: CCK8 assay showing suppression of cell proliferation by piR-2158 overexpression in MDA-MB-231 cells. B: Ki67 staining of MDA-MB-231 cells with or without piR-2158. C: Quantitative analysis of B. D: Wound healing assay demonstrating inhibition of cell migration by piR-2158 overexpression in MDA-MB-231 cells. E: Quantitative analysis of D. F: Transwell assay showing inhibition of cell invasion by piR-2158 overexpression in MDA-MB-231 cells. G. Quantitative analysis of F. H, I: Downregulation of EMT markers Slug, Vimentin, Fibronectin and ZEB1 at the levels of mRNA (H) and protein (I) in the piR-2158-overexpressing MDA-MB-231 cells. J. Quantitative analysis of I. K, L: Downregulation of cell stemness genes KLF4, NANOG, SOX2 and OCT4 at the levels of mRNA (K) and protein (L) in the piR-2158-overexpressing MDA-MB-231 cells. M. Quantitative analysis of L. Data are presented as the mean ± SEM (n = 3). * p < 0.05, ** p < 0.01.

**piR-2158 suppressed cell proliferation, migration, EMT and stemness in human breast cancer.** In order to determine the function of piR-2158 in breast cancer, a triple-negative breast cancer cell line MDA-MB-231 and luminal breast cancer cell line MCF-7 were transfected with piR-2158 or anti-piR-2158, respectively, followed by a series of functional assays including cell proliferation, migration, invasion, EMT and CSC analysis. As a result, overexpression of piR-2158 in both cell lines (Figure S3) significantly reduced cell proliferation (Figure 2A, Figure S4A), ki67 expression (Figure 2B-2C, Figure S4B-S4C) and cell migration and invasion (Figure 2D-2G). In addition, the EMT markers including Vimentin, Fibronectin, Slug and ZEB1, and the CSC markers including KLF4, NANOG, SOX2 and OCT4 were suppressed by piR-2158 (Figure 2H-2M, Figure S4D-S4E). Consistently, knockdown of piR-2158 in either MDA-MB-231 (Figure S5A-S5H) or MCF-7 (Figure S6A-S6F) cells promoted the cell proliferation, migration, EMT, and stemness as well.

**Figure 3:**
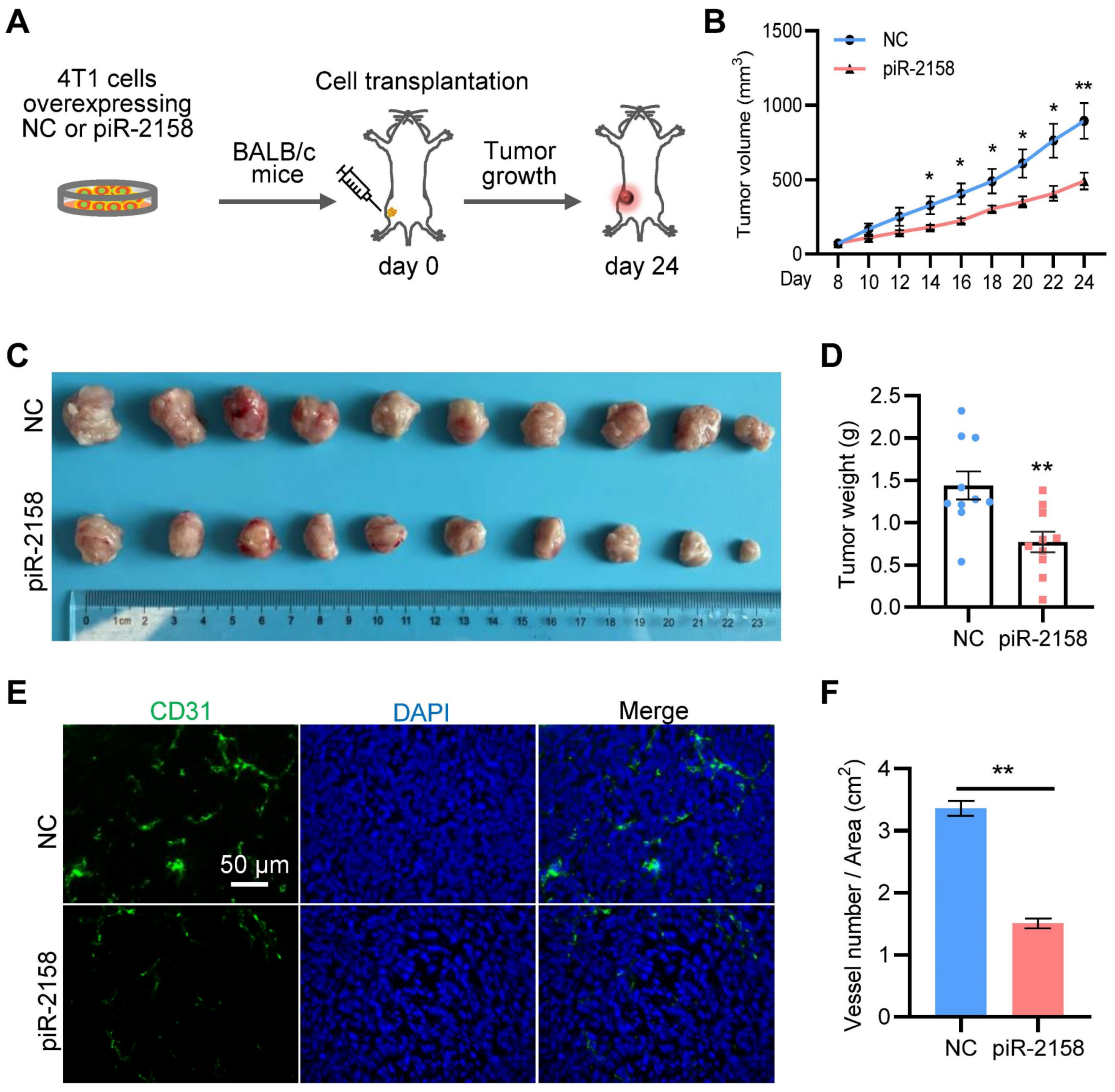
** piR-2158 suppressed mammary tumor growth and angiogenesis *in vivo*.** A: Schematic procedure for preparing the xenograft mouse model with breast cancer. B: Tumor growth curves showing inhibition of tumor growth by piR-2158 overexpression *in vivo*. C: Tumor Images from the mice in A. D: Quantitative analysis of the tumor weights in C (n = 10). E: Immunofluorescence staining of CD31 in the tumor tissues with or without overexpression of piR-2158. F: Quantitative analysis of E (n = 3). Data are presented as mean ± SEM. * p < 0.05, ** p < 0.01.

**piR-2158 suppressed tumor growth and angiogenesis in a xenograft mouse model with breast cancer.** In order to determine the function of piR-2158 *in vivo*, we constructed breast cancer 4T1 cells stably overexpressing piR-2158 using Lentivirus vector LV3 carrying GFP (Figure S7). A xenograft BALB/c mouse model with breast cancer was developed by transplantation of 4T1 cells with or without overexpressing piR-2158 (Figure 3A). The tumor growth curve indicated significant suppression of tumor growth by piR-2158 (Figure 3B), which was further confirmed by the tumor volumes and tumor weights (Figure 3C-3D). In view of the importance of angiogenesis during tumorigenesis, we performed immunofluorescence staining of CD31 which is widely considered as a specific marker of vascular endothelial cells. As shown in Figure 3E and 3F, there were much less CD31+ cells within the tumors derived from the piR-2158-overexpressing 4T1 cells, compared to controls, suggesting suppression of angiogenesis by piR-2158 *in vivo*.

**Figure 4:**
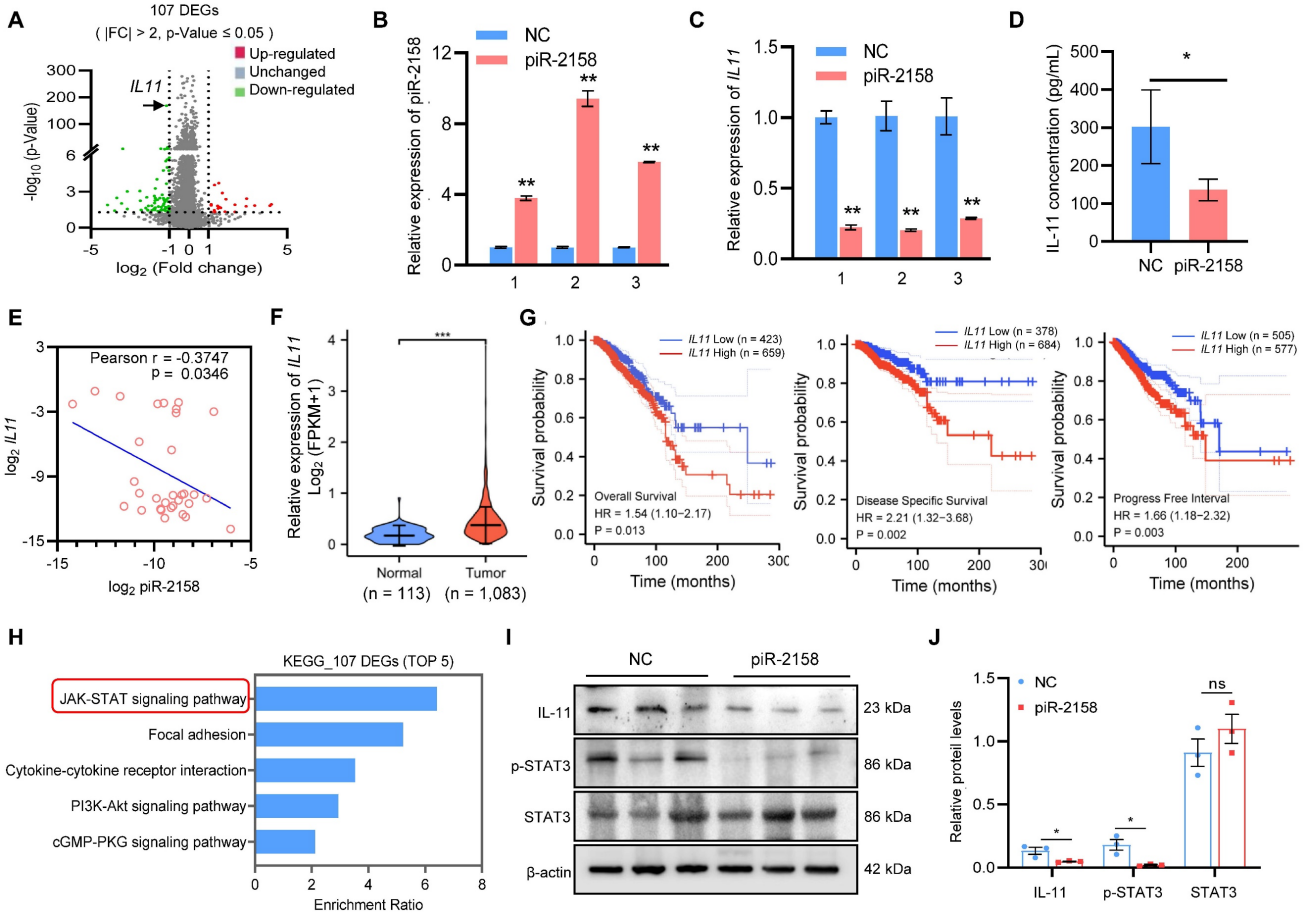
** piR-2158 suppressed *IL11* expression and inactivated JAK-STAT signaling in breast cancer.** A: Volcano plot showing the different expression genes (DEGs) regulated by piR-2158 in MDA-MB-231 cells, in which *IL11* was significantly downregulated. B: Overexpression of piR-2158 in MDA-MB-231 cells in 3 independent experiments. C: QRT-PCR analysis validated downregulation of *IL11* by piR-2158 in MDA-MB-231 cells in B. D: ELISA analysis showing decreased level of secretory IL-11 in the supernatants of piR-2158-overexpressing MDA-MB-231 cells. E: A negative correlation between the expression levels of piR-2158 and *IL11* in tumor tissues from breast cancer patients (n = 32). F: TCGA database showing upregulation of *IL11* in human breast cancer tumors (n = 1,083). G: Negative correlations between the *IL11* expression levels and overall survival, disease specific survival and progression free survival in breast cancer (n = 1,083). H: KEGG pathway analysis showing the top 5 signaling pathways regulated by piR-2158. I and J: Western blot analysis showing downregulation of IL-11 and p-STAT3 by piR-2158 in the mammary tumors from mice. Total STAT3 did not show change. β-actin served as a loading control. Data are presented as the mean ± SEM (n = 3 unless otherwise indicated). * p < 0.05, ** p < 0.01.

**piR-2158 suppressed *IL11* expression and inactivated JAK-STAT signaling.** In order to determine the mechanisms through which piR-2158 inhibits mammary tumorigenesis, we applied RNA-seq analysis to MDA-MB-231 cells with or without overexpression of piR-2158. Total RNAs (n = 3 in each group) were end linked with adapters for amplification. RNA-seq analyses were applied using BGISEQ sequencing platform. The abundance of each gene was quantified as TPM (Transcripts per million) value. The absolute value of fold change (FC) greater than 2 and p-value less than 0.05 were set for cutoff. 107 differently expressed genes (DEGs) were identified including *interleukin 11* (*IL11*) (Table S4, Figure 4A). Suppression of *IL11* expression by piR-2158 was further verified by additional independent analyses (Figure 4B, 4C). Enzyme linked immunosorbent assay (ELISA) of supernatants from MDA-MB-231 cells demonstrated decrease of the secreted IL-11 levels by piR-2158 (Figure 4D). A negative correlation between the piR-2158 and *IL11* levels was observed in the tumors we collected from breast cancer patients (Figure 4E). Further analysis using TCGA database indicated upregulation of *IL11* in breast cancer tumors (Figure 4F). Negative correlations between the *IL11* expression levels and overall survival, disease specific survival and progression free survival were observed in breast cancer patients (Figure 4G). Meanwhile, KEGG analysis of the piR-2158-regulated genes in Figure 4A indicated their enrichment in five main signaling pathways including JAK/STAT (Figure 4H). GSEA analysis indicated their enrichment in Wnt signaling, EMT, and stem cell pluripotency pathway as well (Figure S8). The xenograft tumor tissues in Figure 3C were applied for validation of IL-11 and STAT3. As shown in Figure 4I and 4J, piR-2158 significantly decreased the protein levels of IL-11 and phosphorylated STAT3 in mammary tumors, but did not change the levels of total STAT3. Notably, IL-11 has been reported to trigger JAK/STAT signaling in regulation of human cancer 17. Activation of STAT3 induced angiogenesis 18,19 and promoted the self-renewal capacity of CSCs 20,21.

**Figure 5:**
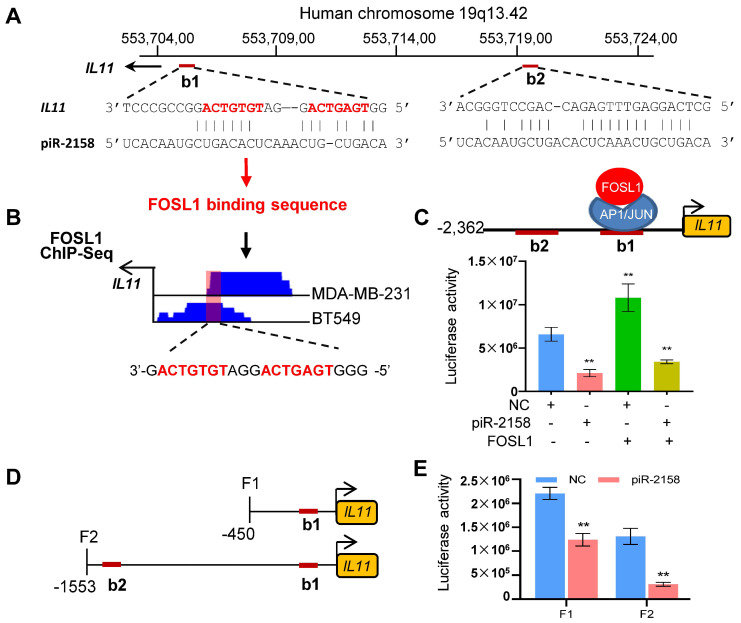
** piR-2158 inhibited *IL11* expression by competing with transcriptional factor FOSL1 to bind the promoter of *IL11*.** A: BLAST analysis identified two potential binding sites (b1 and b2) of piR-2158 in the promoter region of *IL11*, in which the first binding site b1 also belongs to the binding site of FOSL1 (indicated with red color of font). B: ChIP-seq analyses of FOSL1 in two breast cancer cell lines (MDA-MB-231 and BT549) both indicated a binding peak in the b1 region of *IL11* promoter. C: Luciferase reporter assays confirmed suppression of *IL11* promoter by piR-2158, while activation of *IL11* promoter by FOSL1. piR-2158 overexpression attenuated FOSL1-induced *IL11* expression. D: Schematic representation of the two truncated luciferase reporters of *IL11* promoter carrying with b1 (F1) or both b1 and b2 (F2). E: Luciferase reporter assays showing suppression of *IL11* promoter activity by piR-2158 through both b1 and b2 sites. Data are presented as the mean ± SEM (n = 3). ** p < 0.01.

**piR-2158 inhibited *IL11* transcription by competing with FOSL1.** To determine the mechanism for piR-2158 to suppress *IL11*, the Basic Local Alignment Search Tool (BLAST) was applied. Two potential binding sites of piR-2158 were identified in the promoter region of *IL11* (Figure 5A). It aroused our interest that one of the two sites is also the binding site of transcription factors FOS Like 1 (FOSL1) (Figure 5B). In order to determine the relationship between piR-2158 and FOSL1 in control of the transcription of *IL11*, a luciferase reporter driven by the *IL11* promoter was constructed, then cotransfected with piR-2158 mimic and/or *FOSL1*-overexpressing vector. As shown in Figure 5C, piR-2158 alone inhibited the luciferase activity. FOSL1 alone promoted the luciferase activity. Notably, the FOSL1 induction of *IL11* was attenuated by piR-2158 overexpression (Figure 5C), demonstrating a competitive regulation of *IL11* expression by piR-2158 and FOSL1, which was further confirmed in MDA-MB-231 cells with or without transfection with piR-2158 and/or FOSL1 (Figure S9). In order to determine the contribution of the two binding sites of piR-2158 in suppression of *IL11*, we subsequently constructed two truncated luciferase reporters covering the first binding site (F1) or the two bindings sites (F2) (Figure 5D). We transfected the two reporters into 293T cells with piR-2158, respectively. As a result, both F1 and F2 vectors showed inhibition by piR-2158, suggesting both of the binding sites involved in suppression of *IL11* by piR-2158 (Figure 5E).

**Figure 6:**
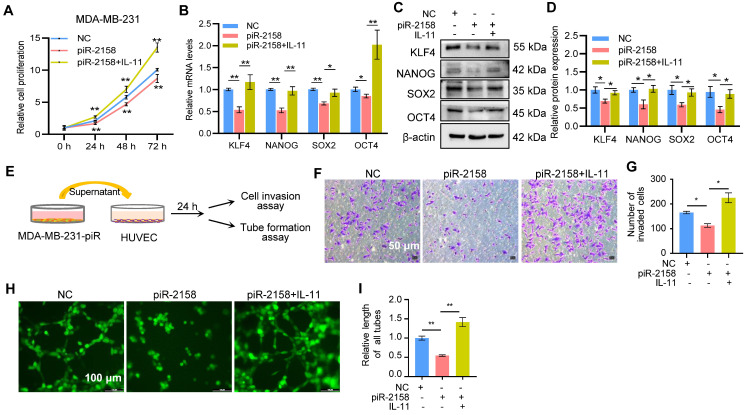
** Exogenous IL-11 rescued the tumor suppression phenotype induced by piR-2158.** A: CCK8 assay showing rescued cell proliferation by addition of exogenous recombinant human IL-11 into the culturing medium of piR-2158-overexpressing MDA-MB-231 cells. B, C: Gene expression analyses demonstrated rescue of the expression of stemness genes KLF4, NANOG, SOX2 and OCT4 at both mRNA (B) and protein (C) levels by addition of exogenous IL-11. D: Quantitative analysis of C. E: Schematic representation of treatment of HUVECs with the conditioned medium (CM) derived from piR-2158-overexpressing MDA-MB-231 cells. F: Transwell assays of HUVECs demonstrating inhibition of cell invasion by piR-2158-CM, which was rescued by addition of exogenous IL-11. G: Quantitative analysis of F. H: HUVECs tube formation assay demonstrated inhibition of capillary-like structure formation by piR-2158-CM, which was rescued by addition of exogenous IL-11. I: Quantitative analysis of H. Data are presented as the mean ± SEM (n = 3). * p < 0.05, ** p < 0.01.

**Exogenous IL-11 rescued the piR-2158-induced phenotypes.** In order to clarify the function of *IL11* signaling in suppression of CSCs and angiogenesis by piR-2158 in breast cancer, we added exogenous recombinant human IL-11 back into the culturing medium of piR-2158-overexpressing MDA-MB-231 cells, followed by functional assays including cell proliferation, cell stemness, transwell invasion and angiogenesis. As shown in Figure 6A-6D, addition of exogenous IL-11 increased the cell proliferation (Figure 6A), and rescued the expression of stemness genes KLF4, NANOG, SOX2 and OCT4 at the levels of both mRNA and protein (Figure 6B-6D). Application of the conditioned medium derived from piR-2158-overexpressing MDA-MB-231 cells to human umbilical vein endothelial cells (HUVEC) significantly suppressed both cell invasion and tube formation, which were rescued by addition of exogenous IL-11 (Figure 6E-6I).

In addition to the heterotypic regulation of angiogenesis by piR-2158, we further determined whether piR-2158 directly affects HUVECs. As shown in Figure S10A-S10C, piR-2158 overexpression in HUVECs reduced the expression of *IL11* and suppressed tube formation.

**Figure 7:**
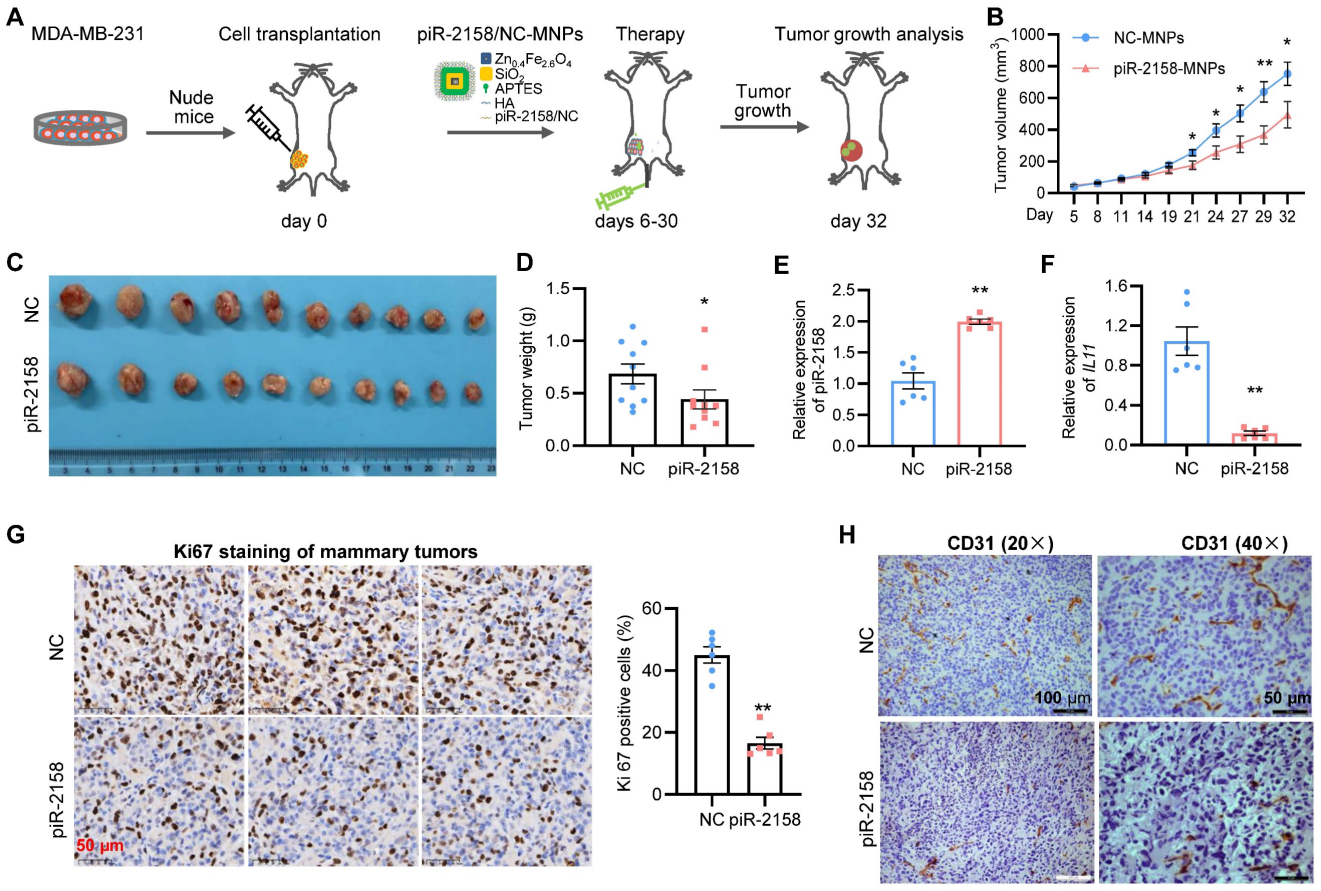
** Tumor-targeted therapy of breast cancer with nanotechnology-based piR-2158.** A: Schematic representation of treatment of mammary tumor-burden mice with hyaluronic acid (HA)-coated, piR-2158-preloaded magnetic nanoparticles (MNPs). B: Tumor growth curves showing decreased tumor growth rate in the mice treated with piR-2158-MNPs (n = 10). C: Tumor Images from the mice in A. D: Quantitative analysis of the tumor weight in C (n = 10). E, F: QRT-PCR analysis demonstrating upregulation of piR-2158 (E) and downregulation of *IL11* (F) in mammary tumors from the mice treated with piR-2158-MNPs, compared to NC group (n = 6). G: Immunohistochemical staining of ki67 in mammary tumors showed inhibition of cell proliferation by piR-2158-MNPs *in vivo* (n = 6). H: Immunohistochemical staining of CD31 in the mammary tumors showed decreased angiogenesis upon treatment with piR-2158-MNPs (n = 3). Data are presented as mean ± SEM. * p < 0.05, ** p < 0.01.

**Therapeutical effect of piR-2158 nanoparticles on breast cancer**. In order to determine the therapeutical effect of piR-2158 on breast cancer, a xenograft model of breast cancer was developed by transplanting MDA-MB-231 cells into immunodeficient nude mice, followed by tail vein injection of the hyaluronic acid (HA)-coated and piR-2158-preloaded magnetic nanoparticles (MNPs) (Figure 7A). It was a dual mammary tumor-targeting drug delivery system by utilizing the HA-CD44 affinity and magnetic attraction. As a result, administration of piR-2158-MNPs significantly suppressed mammary tumor growth in the mice according to the measurements of tumor growth curve (Figure 7B), tumor volume (Figure 7C) and tumor weight (Figure 7D). Higher levels of piR-2158 and lower levels of* IL11* in the piR-2158-MNPs-treated tumors were confirmed, compared to controls (Figure 7E and 7F). Inhibition of cell proliferation and angiogenesis by piR-2158-MNPs were confirmed by immunohistochemical staining of ki67 and CD31 in these mammary tumors, respectively (Figure 7G-7H).

## Discussion

**Figure 8:**
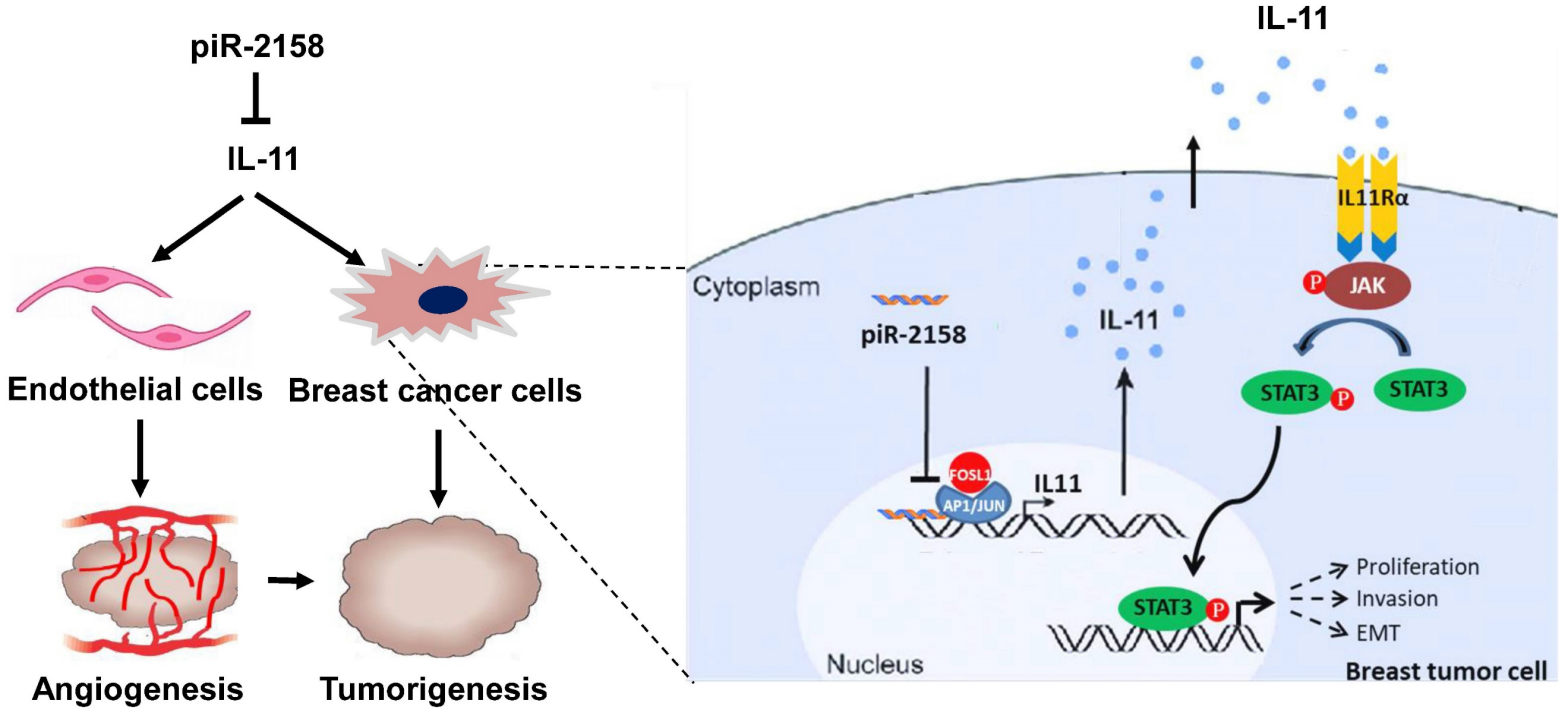
** Schematic representation of the working model in which piR-2158 competed with AP-1 transcription factor subunit FOSL1 to suppress *IL11* expression and IL-11 secretion, thereby inactivating JAK-STAT3 signaling to inhibit tumorigenesis in breast cancer.** Meanwhile, piR-2158-IL11 targeted endothelial cells to inhibited angiogenesis as well.

The major clinical challenges for breast cancer include tumor relapse, distant metastasis and drug resistance, which were all related with CSCs. Development of effective therapeutic strategies targeting CSCs will lead to a new era in the fight against cancer. Involvement of non-coding RNAs in regulation of breast cancer and breast CSCs has been well demonstrated, including miRNAs 22, piRNAs 3 and lncRNAs 23. Aberrant expression of piRNAs in human breast cancer cells has been reported in our previous studies 5,6. Here we identified piR-2158 as a tumor suppressor in breast cancer by regulating cancer cell stemness and angiogenesis in mammary tumors (Figure 8). Both *in vitro* and *in vivo* assays demonstrated the promise of piR-2158 as a therapeutic target in breast cancer.

piR-2158, also known as DQ572892, piR-2980, piRNA-21067, piR-41004, piR-3200, piR-34003 by different databases, is located at the minus chain of chromosome 16. piR-2158 regulation of stem cells has been reported in germline 1 and mesenchymal stem cells (MSCs) 7. There was only one publication in the literature about piR-2158 regulation of human cancers that hypoxia-regulated piR-2158 predicted tumor recurrence and recurrence-free survival in the lung cancer patients with hypoxic tumors 8. piR-2158 has been detected in breast cancer cells by our previous study 5 and the literature 24. However, the regulatory function and mechanisms of piR-2158 in breast cancer remain unclear until our current study demonstrating piR-2158 inhibition of breast cancer by targeting IL11-STAT3 signaling.

Epigenetic regulation is a common mechanism for piRNAs in germ cells, and tumors as well. For example, piR-651 promoted cell proliferation and migration in breast cancer by suppressing Pten *via* recruiting DNA methyltransferase 1 (DNMT1) to its promoter region 25. piR-823 activated Wnt signaling and induced stemness in the luminal subtype of breast cancer cells by increasing DNA methylation of gene *adenomatous polyposis coli* (*APC*) 6. piR-021285 induced cellular invasion in breast cancer by increasing DNA methylation of *ARHGAP11A* 26. The current study reveals a novel regulatory mechanism through which piR-2158 involved in the interaction between AP-1 transcriptional complex and the promoter of *IL11* in human breast cancer cells.

FOSL1 (also named FRA-1) is one of the main AP-1 family transcription factors with diverse functions. FOSL1 induces EMT and carcinogenesis 27,28. IL-11, as a pleiotropic cytokine, plays important roles in a number of cancers including colon and breast cancer. IL-11 induction by oxidative stress *via* FOSL1 signaling has been reported by Nishina *et al* 29. In addition, oxidative stress in inflamed intestinal biopsies induced *FOSL1* gene expression, thereby promoting the expression of *IL11* in ulcerative colitis patients 30. Here we demonstrated *IL11* inhibition by piR-2158 *via* competing with FOSL1 in breast cancer cells. The literature has demonstrated activation of STAT3 by IL-11 in carcinogenesis. IL-11 regulates endometrial cancer cell adhesion and migration *via* upregulating the phosphorylated (p)-STAT3 protein abundance 31. Activation of STAT3 signaling by IL-11 in breast cancer was recently reported to promote tumor invasion and metastasis 32.

Chemo therapy and immunotherapy, as two most popular approaches in treatment of cancer patients 33, are facing challenges of cancer cell adaptation-induced drug resistance and immune evasion 34. Emerging evidence shows promise of biomaterial-based nanoparticles for addressing these challenges in both pre-clinical and clinical studies 35,36. Conjugations of ligands including peptides, antibodies, or nucleic acids to the surface of nanoparticles have been widely demonstrated to deliver drugs to the target tumor cells. For example, Jin *et al*. fabricated Poly-(D, L-lactide-co-glycolide) (PLGA)-based nanoparticles coated with human cancer cell membrane fractions to target cancer cells by interacting with membrane-associated receptors CXCR4 and CD44 37. The pre-clinical and clinical applications of engineered nanoparticles incorporating chemotherapeutic drugs or bioactive compounds in treatment of breast cancer were well reviewed in the literature 38,39.

The uptake of nanoparticles by breast CSCs is both HA and CD44 dependent. HA specially binds and interacts with the cell surface receptor CD44, leading to cancer cell growth and survival 40. High expression of CD44 has been proved to be one of the most important characteristics for breast CSCs 9. In the current study, HA-coated and piR-2158-preloaded MNPs were applied to treat the xenograft model of breast cancer, resulting in significant therapeutic effects, which holds the potential to develop novel therapeutic strategies on the basis of the piRNA regulation of CSCs. Optimization of nanoparticle vehicles carrying piR-2158 to better target human breast cancer cells or CSCs and maximally avoid the off-target effects will be helpful to move it forward into clinical applications.

## Supplementary Material

Supplementary figures and tables.
