# Supplementary material for: IL11 signaling mediates piR-2158 suppression of cell stemness and angiogenesis in breast cancer
Source: Theranostics. 2023 Apr 17;13(7):2337–49. doi: 10.7150/thno.82538 (PMC10157741; doi:10.7150/thno.82538)

Supplemental Figure Legends

Figure S1: FACS analysis of ALDH+ breast cancer stem cells from breast cancer patients.

Figure S2: FACS analysis of ALDH+ breast cancer stem cells from breast cancer cell MCF-7.

Figure S3: Confirmation of overexpression of piR-2158 in MDA-MB-231 (A) and MCF-7 (B) breast cancer cells upon piRNA mimic transfection. Data are presented as mean ± SEM. ** $p < 0.01$.

Figure S4: piR-2158 overexpression decreased cell proliferation, EMT and cell stemness in MCF-7. A-C: Cell proliferation analysis by CCK8 assay (A) and ki67 staining (B and C) in MCF-7 cells with or without piR-2158 overexpression. D: QRT-PCR analysis of stem cell genes KLF4, NANOG, SOX2 and OCT4 in MCF-7 cells transfected with piR-2158 mimic or control. E: QRT-PCR analysis of EMT markers Slug, Vimentin, Fibronectin and ZEB1 in MCF-7 cells transfected with piR-2158 mimic or control. Data are presented as the mean ± SEM (n = 3). * $p < 0.05$, ** $p < 0.01$.

Figure S5: Knockdown of piR-2158 in MDA-MB-231 cells induced the cell proliferation, migration and cell stemness. A: Validation of piR-2158 knockdown by its antisense inhibitor in MDA-MB-231 cells. B-D: Cell proliferation analysis by CCK8 assay (B) and ki67 staining (C and D) in MDA-MB-231 cells with or without knockdown of piR-2158. E: Wound healing assay in MDA-MB-231 cells with or without knockdown of piR-2158. F: Quantitative analysis of E. G: QRT-PCR analysis of EMT markers Slug, Vimentin, Fibronectin and ZEB1 in MDA-MB-231 cells with or without knockdown of piR-2158. H: QRT-PCR analysis of stem cell genes KLF4, NANOG, SOX2 and OCT4 in MDA-MB-231 cells with or without knockdown of piR-2158. Data are presented as the mean ± SEM (n = 3). * $p < 0.05$, ** $p < 0.01$.

Figure S6: Knockdown of piR-2158 in MCF-7 cells induced the cell proliferation, EMT and cell stemness. A: Validation of piR-2158 knockdown in MCF-7 cells. B-D: Cell proliferation analysis by CCK8 assay (B) and ki67 staining (C and D) in MCF-7 cells with or without knockdown of piR-2158. E: QRT-PCR analysis of stem cell genes KLF4, NANOG, SOX2 and OCT4 in MCF-7 cells with or without knockdown of piR-2158. F: QRT-PCR analysis of EMT markers in MCF-7 cells with or without knockdown of piR-2158. Data are presented as the mean ± SEM (n = 3). * $p < 0.05$, ** $p < 0.01$.

Figure S7: Confirmation of overexpression of piR-2158 in 4T1 breast cancer cells. Data are presented as mean ± SEM. ** $p < 0.01$.

Figure S8: GSEA analysis of the differently expressed genes (DEGs) indicating their enrichment in the pathways including Wnt signaling, EMT, stem cell pluripotency, etc.

Figure S9: Expression analysis of *IL11* in MDA-MB-231 cells after transfection with piR-2158 and/or FOSL1. Data are presented as mean ± SEM. * $p < 0.05$, ** $p < 0.01$.

Figure S10: piR-2158 suppressed tube formation directly by targeting *IL11* in HUVECs. A: Overexpression of piR-2158 in HUVECs. B: Downregulation of *IL11* by piR-2158 overexpression in HUVECs. C: Representative images of HUVECs tube formation with or without overexpression of piR-2158. Data are presented as mean ± SEM. * $p < 0.05$, ** $p < 0.01$.

Figure S11-S14: The original images of western blots.

Figure S1

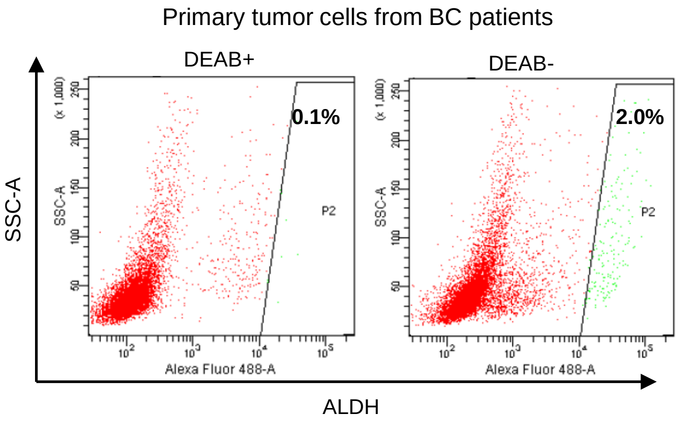

Primary tumor cells from BC patients
DEAB+
DEAB-
0.1%
2.0%
P2
SSC-A
Alexa Fluor 488-A
ALDH

Figure S2

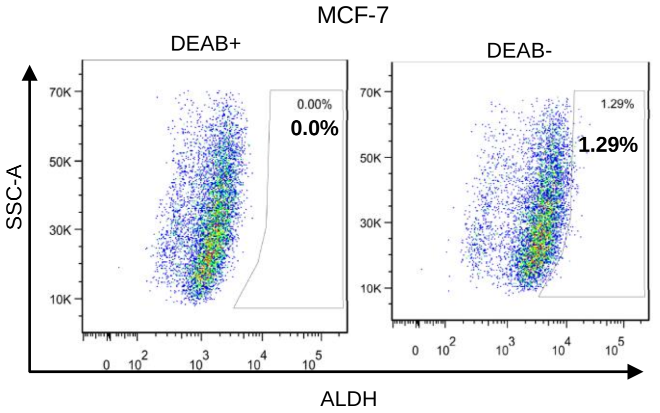
MCF-7
DEAB+
DEAB-
SSC-A
70K
50K
30K
10K
0.00%
0.0%
1.29%
1.29%
0
10^2
10^3
10^4
10^5
ALDH

Figure S3

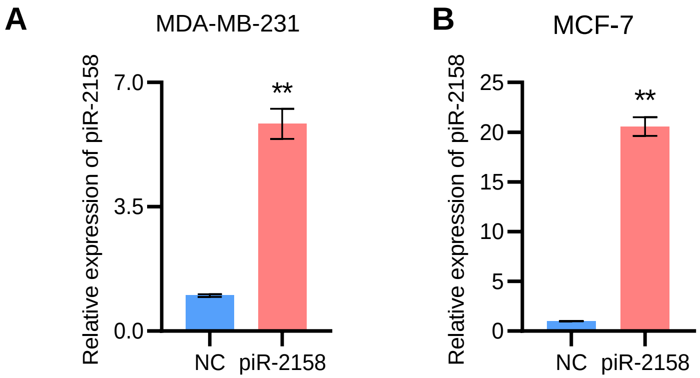
A
MDA-MB-231
Relative expression of piR-2158
7.0
3.5
0.0
**
NC
piR-2158
B
MCF-7
Relative expression of piR-2158
25
20
15
10
5
0
**
NC
piR-2158

Figure S4

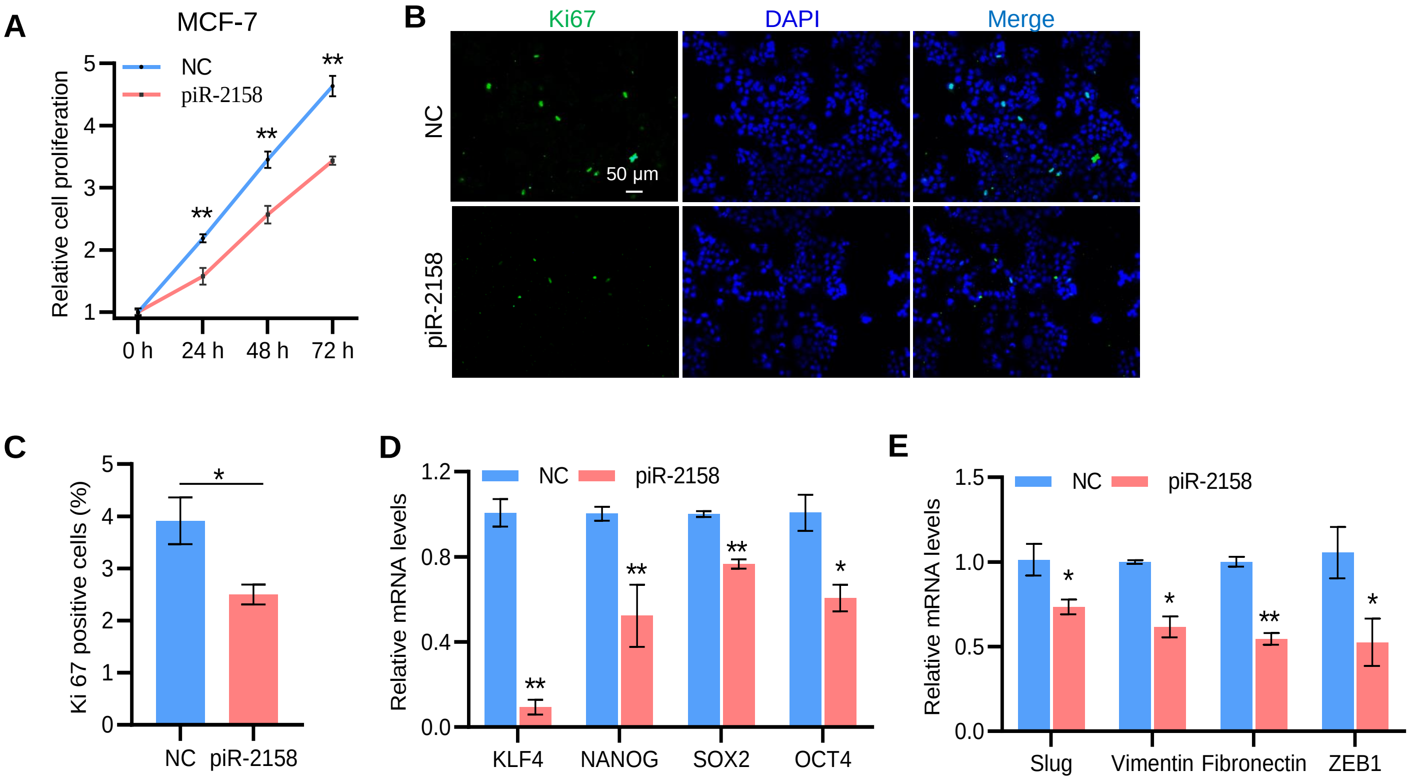

A
MCF-7
NC
piR-2158
Relative cell proliferation
**
0 h
24 h
48 h
72 h
B
Ki67
DAPI
Merge
NC
piR-2158
50 μm
C
Ki 67 positive cells (%)
*
NC
piR-2158
D
Relative mRNA levels
KLF4
NANOG
SOX2
OCT4
E
Relative mRNA levels
Slug
Vimentin
Fibronectin
ZEB1

Figure S5

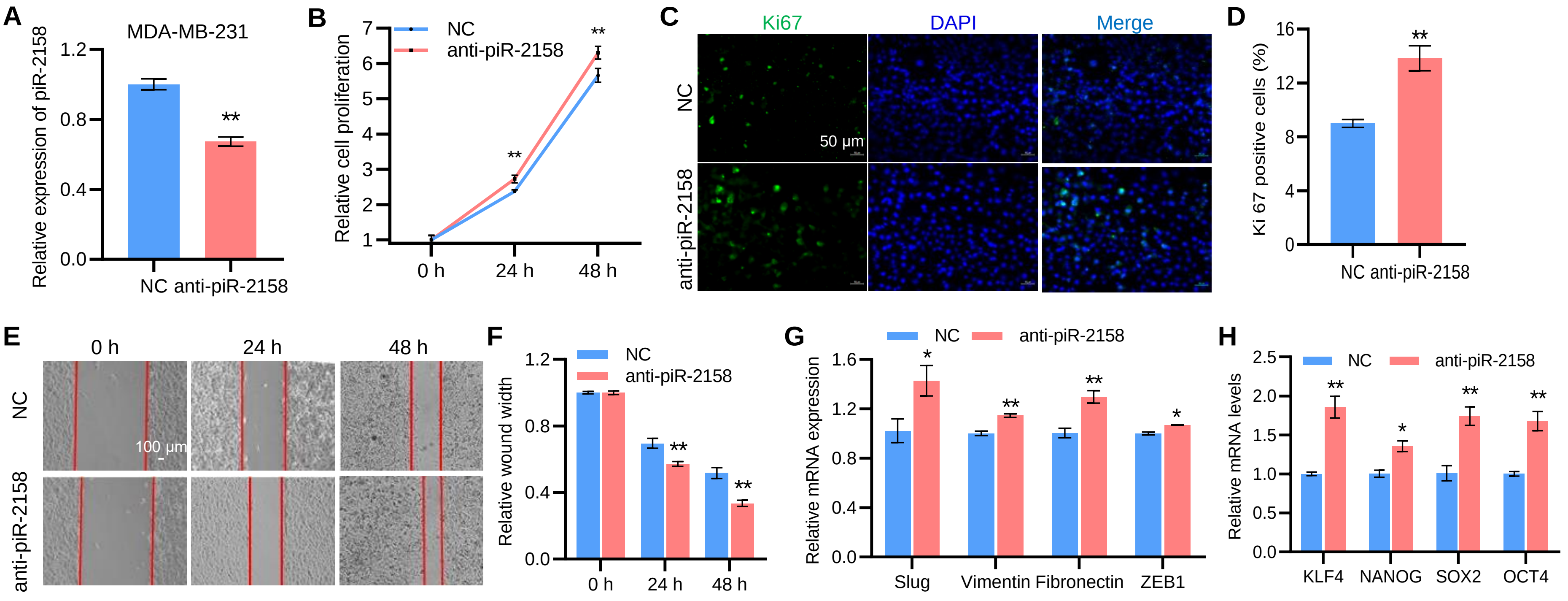

A
MDA-MB-231
Relative expression of piR-2158
1.2
0.8
0.4
0.0
**
NC anti-piR-2158
B
Relative cell proliferation
7
6
5
4
3
2
1
NC
anti-piR-2158
**
**
0 h
24 h
48 h
C
Ki67
DAPI
Merge
NC
anti-piR-2158
50 μm
D
Ki 67 positive cells (%)
16
12
8
4
0
**
NC anti-piR-2158
E
0 h
24 h
48 h
NC
anti-piR-2158
100 μm
F
Relative wound width
1.2
0.8
0.4
0.0
NC
anti-piR-2158
**
**
0 h
24 h
48 h
G
NC
anti-piR-2158
Relative mRNA expression
1.6
1.2
0.8
0.4
0.0
*
**
**
*
Slug
Vimentin
Fibronectin
ZEB1
H
Relative mRNA levels
2.5
2.0
1.5
1.0
0.5
0.0
NC
anti-piR-2158
**
*
**
**
KLF4
NANOG
SOX2
OCT4

Figure S6

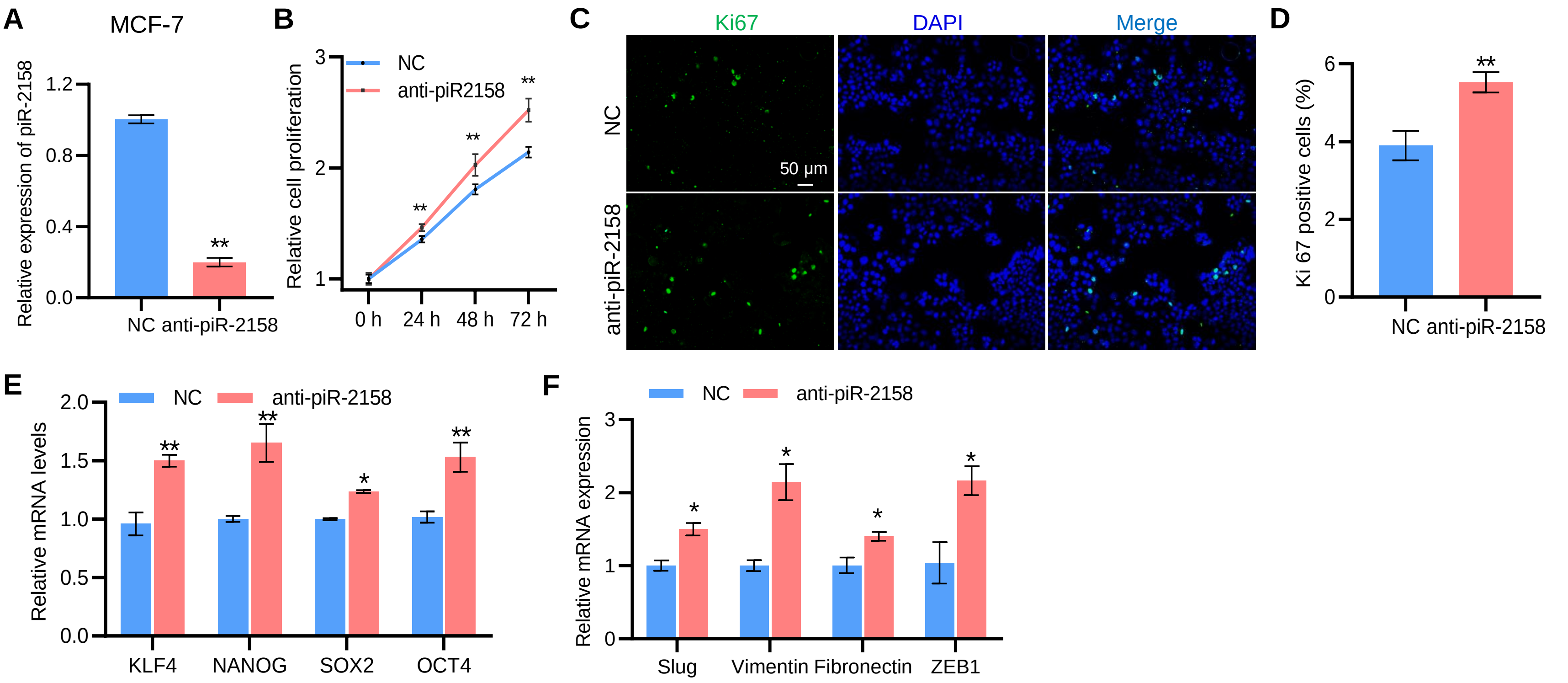

A
MCF-7
Relative expression of piR-2158
1.2
0.8
0.4
0.0
**
NC anti-piR-2158
B
Relative cell proliferation
3
2
1
NC
anti-piR2158
**
0 h 24 h 48 h 72 h
C
Ki67
DAPI
Merge
NC
anti-piR-2158
50 μm
D
Ki 67 positive cells (%)
6
4
2
0
**
NC anti-piR-2158
E
NC anti-piR-2158
Relative mRNA levels
2.0
1.5
1.0
0.5
0.0
**
*
KLF4 NANOG SOX2 OCT4
F
NC anti-piR-2158
Relative mRNA expression
3
2
1
0
*
Slug Vimentin Fibronectin ZEB1

Figure S7

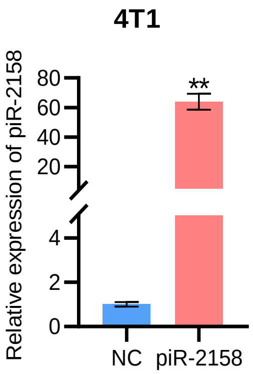
4T1
Relative expression of piR-2158
80
60
40
20
4
2
0
**
NC
piR-2158

Figure S8

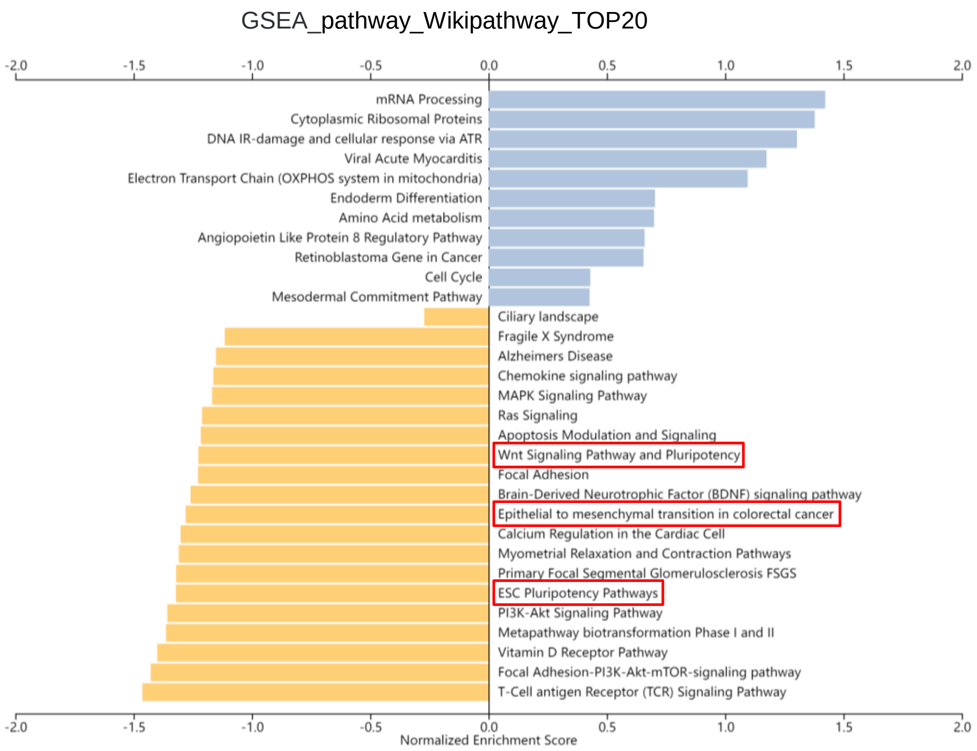

GSEA_pathway_Wikipathway_TOP20
-2.0
-1.5
-1.0
-0.5
0.0
0.5
1.0
1.5
2.0
mRNA Processing
Cytoplasmic Ribosomal Proteins
DNA IR-damage and cellular response via ATR
Viral Acute Myocarditis
Electron Transport Chain (OXPHOS system in mitochondria)
Endoderm Differentiation
Amino Acid metabolism
Angiopoietin Like Protein 8 Regulatory Pathway
Retinoblastoma Gene in Cancer
Cell Cycle
Mesodermal Commitment Pathway
Ciliary landscape
Fragile X Syndrome
Alzheimers Disease
Chemokine signaling pathway
MAPK Signaling Pathway
Ras Signaling
Apoptosis Modulation and Signaling
Wnt Signaling Pathway and Pluripotency
Focal Adhesion
Brain-Derived Neurotrophic Factor (BDNF) signaling pathway
Epithelial to mesenchymal transition in colorectal cancer
Calcium Regulation in the Cardiac Cell
Myometrial Relaxation and Contraction Pathways
Primary Focal Segmental Glomerulosclerosis FSGS
ESC Pluripotency Pathways
PI3K-Akt Signaling Pathway
Metapathway biotransformation Phase I and II
Vitamin D Receptor Pathway
Focal Adhesion-PI3K-Akt-mTOR-signaling pathway
T-Cell antigen Receptor (TCR) Signaling Pathway
Normalized Enrichment Score

Figure S9

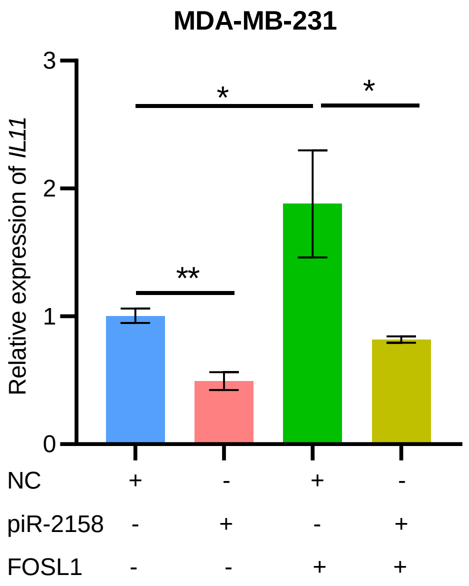
MDA-MB-231
Relative expression of IL11
0
1
2
3
*
*
**
NC + - + -
piR-2158 - + - +
FOSL1 - - + +

Figure S10

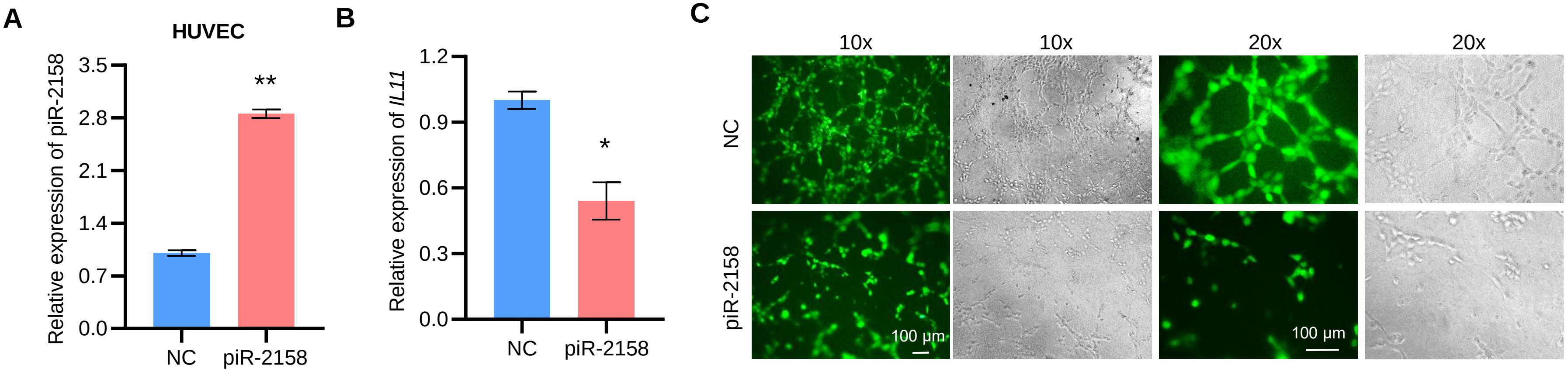
A
HUVEC
Relative expression of piR-2158
3.5
2.8
2.1
1.4
0.7
0.0
**
NC
piR-2158
B
Relative expression of IL11
1.2
0.9
0.6
0.3
0.0
*
NC
piR-2158
C
10x
10x
20x
20x
NC
piR-2158
100 μm
100 μm

Figure S11 Original uncropped images for Figure 2I

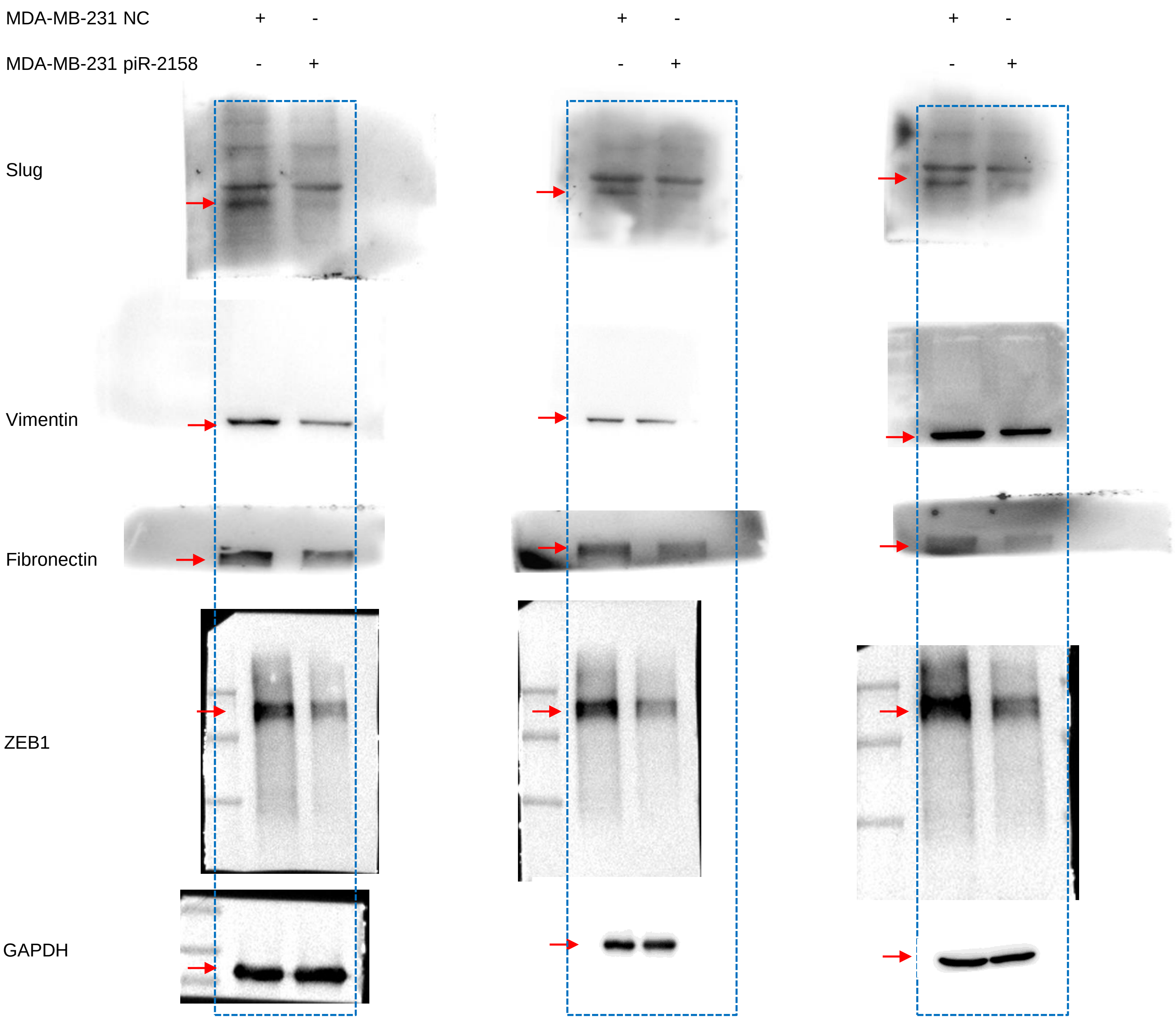

Figure S12 Original uncropped images for Figure 2L

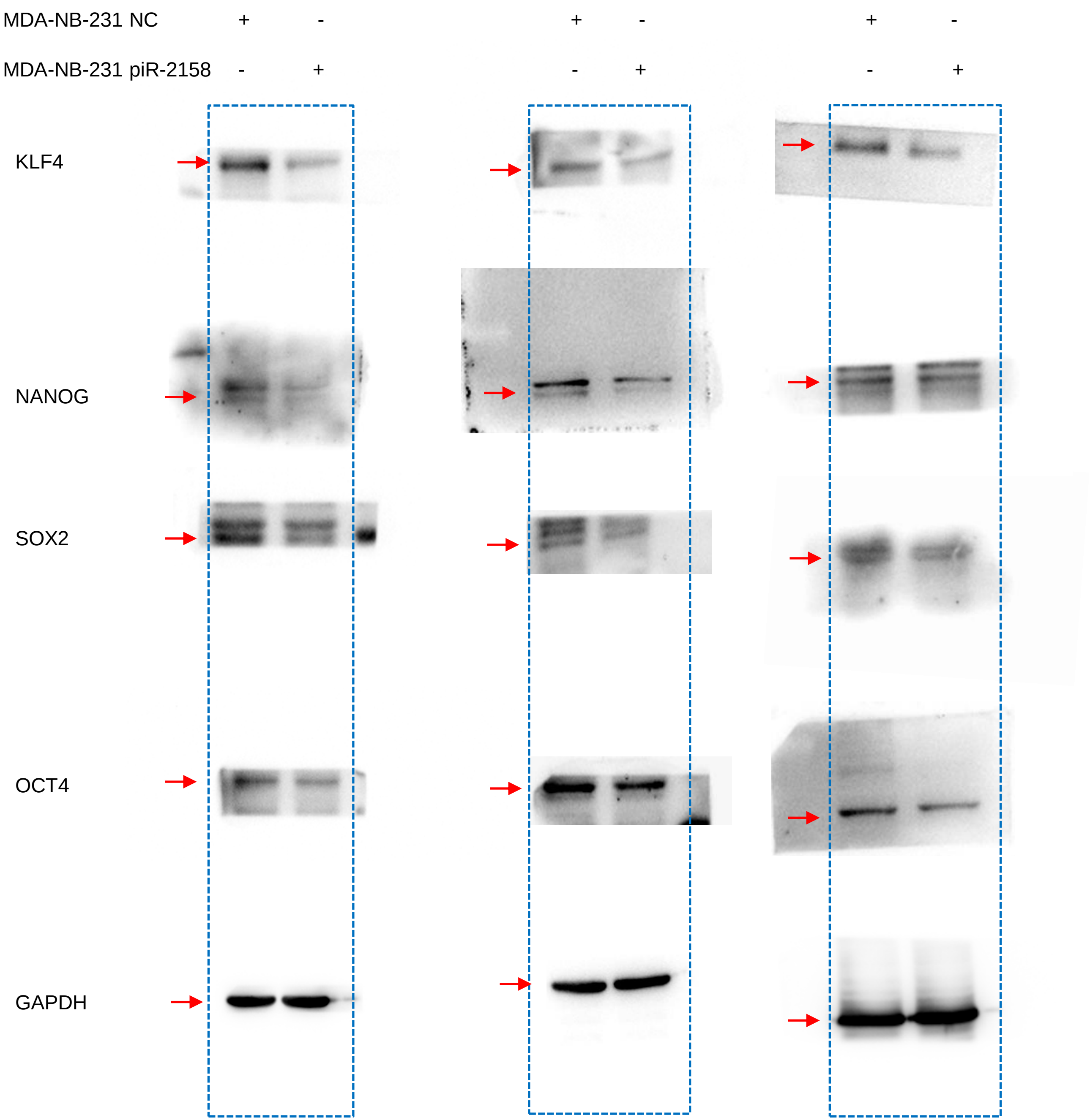

Figure S13 Original uncropped images for Figure 4I

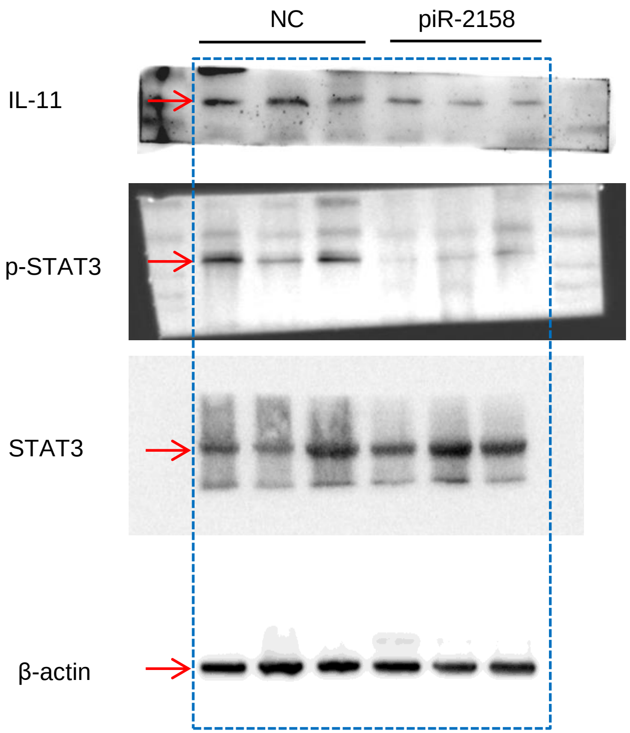

Figure S14 Original uncropped images for Figure 6C

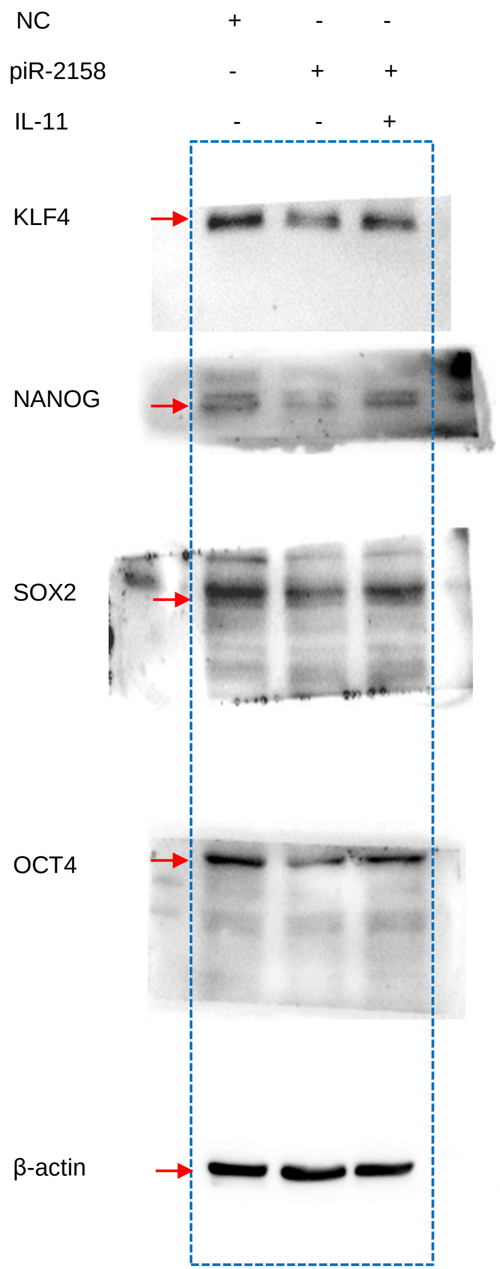

Supplement: Supplementary file 1 — Supplementary figures and tables. [file thnov13p2337s1.zip › Supplemental Figures.pdf]
